# Frequency of Different Dental Irregularities Associated With Cleft Lip and Palate in a Tertiary Care Dental Hospital

**DOI:** 10.7759/cureus.14456

**Published:** 2021-04-13

**Authors:** Noor U Huda, Hazik B Shahzad, Maria Noor, Yaser Ishaq, Malik Adeel Anwar, Muhammad Kashif

**Affiliations:** 1 Department of Oral Biology, Rashid Latif Medical & Dental College, Lahore, PAK; 2 Department of Community Dentistry, Rashid Latif Medical & Dental College, Lahore, PAK; 3 Department of Oral Medicine, Fatima Memorial Hospital College of Medicine & Dentistry, Lahore, PAK; 4 Department of Oral Surgery, Akhtar Saeed Medical & Dental College, Lahore, PAK; 5 Department of Biomedical Engineering, Binghamton University, New York, USA; 6 Department of Oral Pathology, Fatima Memorial Hospital College of Medicine & Dentistry, Lahore, PAK; 7 Department of Oral Pathology, Bakhtawar Amin Medical and Dental College, Multan, PAK

**Keywords:** oral condition, cleft lip, cleft palate, dental anomalies, oral conditions, lahore

## Abstract

Background

Oro-facial clefts (OFCs) are formed due to a combination of genetic factors and environmental factors. Treatment is usually extensive and lasts till adult age. The treatment also includes a large portion of dental rehabilitation.

Objective

This study aims to look at the different dental anomalies associated with OFCs.

Methods

A total of 100 participants with OFCs were randomly selected from Clap centre Lahore. They were categorized into cleft lip (CL), cleft palate (CP), and both. Dental anomalies were recorded clinically and family history for OFCs was also recorded.

Results

Out of the 100 participants, 15 had CL only, 37 had a CP, and 48 had both CL and CP. Missing teeth and hypodontia were significantly associated with all OFCs (p-value > 0.05). Supernumerary teeth were only significantly associated with CP (p-value: 0.04). Other dental anomalies were not significant for OFCs.

Conclusion

OFCs in all its three forms are associated with dental irregularities. They can either be missing teeth or extra teeth. There is a strong need for dentists to be a part of the treatment planning of OFCs and to treat dental anomalies alongside the clefts.

## Introduction

Craniofacial growth and maturity is a complex process during prenatal embryonic development. Minor irregularities during embryonic development can result in major congenital malformations of oro-facial region such as cleft lip and palate [1]. Oro-facial clefts (OFCs) are structural defects of the face and oral cavity which are responsible for severe aesthetic malformations and abnormal functioning of oral cavity [2]. Within OFCs, the main categories are cheiloschisis/cleft lip (CL) and palatoschisis/cleft palate (CP), and submucous cleft palate /soft palate cleft (SMCP). OFCs may also present as a combination of these clefts such as cleft lip and palate (CLP). Epidemiological surveys have demonstrated that OFCs are the most common anomaly among birth defects in children [3]. It has been documented that one in every six hundred babies born is affected with OFCs. Variation in different oral clefts is highly gender-specific as CP is commonly seen among females. On the other hand, CLP is more common among males [4].

As a multifactorial disease, genetic factors and environmental factors both play a role in the development of clefts [1]. Lip is formed at the 4th and 7th week while palate develops at the 5th to 8th week of intrauterine life [5]. A number of transcription factors along with signaling molecules and genes are responsible for controlling this process. Changes in the regulation process of these transcription factors and gene mutations can easily lead to change in DNA sequence which further account for the many congenital abnormalities of orofacial region [1]. Several environmental factors are also associated with the incidence of developing cleft lip or palate [6]. Toxic effects of drugs such as hydantoin, sodium valproate, trimethadione are well evident for the development of OFCs. Child-bearing women suffering from diseases such as diabetes mellitus, and phenylketonuria are also susceptible to having babies with different facial clefts [7]. Complications during early pregnancy including amniotic rupture are also a factor for developing OFCs [3]. Avoidance of factors such as poor living habits, chemical factors, age, and poor health status of women during pregnancy is key for normal craniofacial development [6]. OFCs can also occur as isolated birth defects due to the influence of environmental factors such as tobacco, folic acid deficiency, and certain drug interactions. Syndromic clefts on the other hand are due to a combination of genetic and environmental factors [8]. The factors affecting non-syndromic clefts are not fully understood, however, OFCs are mostly associated with both syndromic and genetic conditions [3,9].

Primary and permanent dentitions may be affected equally because of OFCs. Furthermore, dentition on the affected cleft side is compromised frequently [10]. It has been recorded that 96.7% OFC patients develop at least one dental deformity [11]. Dental anomalies in the cleft region suggest that clefts do not affect dentition generally, but rather they affect dentition locally as in on a particular tooth only [10]. The prevalence of dental anomalies is dependent upon the severity of the existing cleft [12]. Complete absence of a tooth is well appreciated in the quadrant which is affected by the cleft. A specific tooth is usually affected in OFCs which may include premolars, lateral incisors or canines. Lateral incisors are the most commonly affected tooth in OFC dentition [13]. Agenesis is characteristically seen in unilateral cleft lip and palate [13]. Contrarily, impactions are more commonly appreciated in patients with CLP in the anterior compartment of the oral cavity including the premolar area [11]. Patients with cleft show different dental defects such as hypo-mineralization of enamel, supernumerary teeth, peg-shaped or fused teeth, unilateral crossbite, and crowding [14].

Despite, a vast range of treatments for craniofacial abnormalities, they exhibit a high psychosocial impact on patients. These psychosocial impacts are due to the presence of facial disfigurement along with dental malformation. Due to new approaches to craniofacial rehabilitation, the healthcare community can assess facial as well as dental abnormalities at the fetal stage via fetal dental panorama [15]. The main objective of this study is to comprehend dental anomalies associated with specific clefts so, that prior treatment planning of dental deformity can be assessed and managed before eruption of teeth.

## Materials and methods

This cross-sectional research was conducted at the Clap Centre, Lahore. Clap Centre is a dedicated free-of-cost treatment centre for OFCs covering all of the Punjab province. It was completed over a period of five months from June 2019 to October 2019. Ethical permission was granted by the ethical review committee at Rashid Latif Dental College (Ref No RLDC/2197/2020). Verbal consent was obtained from each participant before the questionnaire. Participants were informed about the benefits of the study and were informed about their voluntary participation along with data protection.

The sample size was calculated based on the prevalence rate in Pakistan for CLP. It is estimated that there are 1.9 cases of CLP in every 1,000 births [16]. With a precision value of 0.05 and a 1.96 level of confidence, it was estimated that a sample of 65 participants would be enough to show any significant change. The sample was rounded off to 100 participants for ease and to account for any losses. Inclusion criteria comprised all patients with OFCs visiting Clap Centre, Lahore for treatment purposes. Participants denying consent and patients with any syndrome were excluded. A dentist was trained as an examiner to orally ask and fill in the descriptive form. The examiner was also trained and calibrated against a gold standard for intraoral examinations, using head-light and tongue depressor only.

Children with OFCs were classified into three groups. The respective groups were; children with cleft lip only, children with cleft palate only and children with both cleft lip and cleft palate. The other demographic variables included age and gender. OFC has a genetic or environmental cause and the fetal development stage is not affected by any social demographics (education, employment, etc.).This is the reason they weren't recorded [6]. History of OFCs in parents and grandparents were asked and recorded. The dental anomalies recorded, comprised of Hypodontia type 1 (single missing tooth), Hypodontia type 1 (less than six teeth missing), oligodontia (more than six teeth missing), microdontia (<50% of the expected size), macrodontia (1.5 times larger than normal), Hutchinson incisors (notched incisal edge), supernumerary and malformed teeth (all other malformations). Special care was taken to account for teeth affected by the OFCs only and missing due to caries or any orthodontic reasons were not included in the study. All patients at Clap Centre had Orthopantomogram (OPG) radiographs with them, so it was easy to evaluate for unerupted canines and premolars in young children.

Collected data were entered into statistical software package STATA-14 (STATA Corp, College Station, TX, USA) for further analysis. Chi-squared test was used and a 95% significance level (p-value <0.05) was selected for p-value.

## Results

The final sample size consisted of 100 participants. The sample had more male (56%) participants than female participants. The mean age of the sample was 13.4 years (95% CI 12.4-14.4). Out of the sample, 15 participants had cleft lip only and 37% of the sample had cleft palate only. 48% of the sample had both cleft lip and cleft palate. Looking at family history, 13% of the sample showed a genetic predisposition by having a parent or grand-parent afflicted with OFCs.

**Figure 1:**
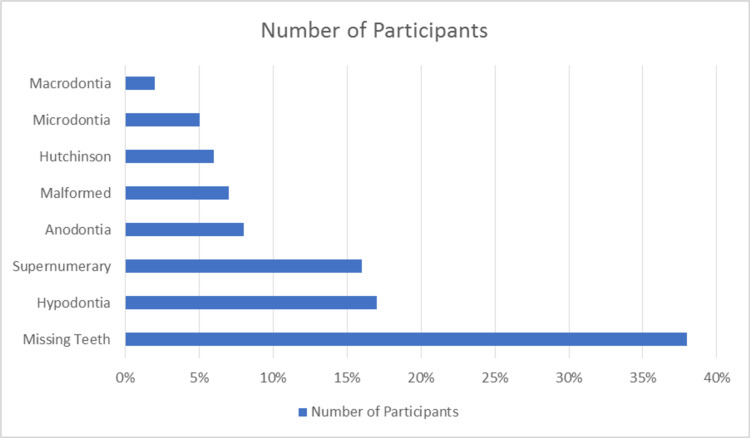
Distribution of dental anomalies in oro-facial cleft patients.

Looking at the dental anomalies, the most common dental anomaly was a single missing tooth. 38% of the sample had one tooth missing. The most common missing tooth was premolar at 19%, followed by lateral incisors (16%). 17% of the sample had more than one tooth missing (hypodontia). Complete anodontia was found in 8% of the sample. Other dental anomalies are mentioned in Figure 1.

Chi-squared and Fisher’s exact test showed significant results for missing teeth and hypodontia. Missing teeth were significantly related to patients with cleft lip, cleft palate, and both cleft lip/palate having significant p-values (p-value >0.01). Hypodontia was also significant for all OFCs. Supernumerary teeth were only significant in a combination of cleft lip and palate with a p-value of 0.04. Hutchinson incisor, microdontia, macrodontia, and malformed teeth were not significantly related to any OFC. All the anomalies with their respective p-values can be seen in Table 1.

**Table 1:** Chi-squared test for dental anomalies in cleft lip/palate patients (n = 100).

Variable	Cleft lip	Cleft palate	Cleft lip and palate
Missing teeth	0.001	0.007	0.000
Hypodontia	0.007	0.031	0.006
Supernumerary	0.06	0.640	0.04
Anodontia	0.49	0.063	0.31
Malformed	0.69	0.297	0.123
Hutchinson Incisor	0.30	0.19	0.68
Microdontia	0.84	0.74	0.92
Macrodontia	0.05	0.54	0.22

## Discussion

This study was conducted with the help of 100 participants with OFCs. It was seen that 15 participants had cleft lip only, 37 had cleft palateonly, and 48 had both cleft lip and cleft palate. Missing teeth and hypodontia were significantly associated with all OFCs. Supernumerary teeth were only significantly associated with both cleft lip and palate (CLP). The existence of dental anomalies with CLP is variable for different ethnic groups [17]. It has been reported in previous research that dental deformities with unilateral cleft lip and palate (UCLP) are more common in the Caucasian population [17]. Similarly, this study conducted on the Asian population of Pakistan showed an increased risk of dental deformities with the presence of OFCs. It has been noted that genetics plays a major role in the development of dental anomalies in patients with cleft and is the main cause for dental anomalies in all ethnicities. It has been documented that CLP is far more common compared to CL and CP alone [17]. In contrast, this study showed a higher incidence of CL and CP individually (64%) rather than the presence of both as CLP (49%). This difference can be justified by the presence of different genetic coding among individuals of two different populations. Genes or transcription factors during normal lip and palate development such as Tbx and Barx can be altered in one population even by some epigenetic factors which are not distinguished in other populations [18]. So, epigenetics and genetics of two different populations justify the presence of more CLP in one and higher CL/CP individually in another population. It has been observed previously that females are mostly affected with unilateral clefts compared to males [19]. On the other hand, in this study more male population (56%) was presented with OFCs compared to the female population (44%). Representation of more male communities with clefts and dental deformities might be due to the fact that in this community people are more concerned for the better living of males as compared to females.

The type of dental anomalies in patients with cleft is not gender-specific [20]. Similarly, this study also agrees with previous literatures demonstrating that all kinds of dental anomalies are widely spread over both genders with OFCs [11]. It is not published yet that a specific OFC may present with a specific dental anomalies. Missing teeth are most common among patients with clefts. These missing teeth may be due to agenesis or due to the impaction of the missing teeth. Impacted teeth can be treated and brought back into the dentition via orthodontic appliances. However, agenesis cannot be overcome and may require rehabilitation using a dental prosthesis at the treatment stage. It has been already reported that missing teeth are more common at the cleft side [11]. Similarly, dental deformities such as hypodontia, supernumerary teeth, microdontia and macrodontia were seen more frequently on the cleft side. This statistic shows that the deformed oral cavity or face does not allow teeth to erupt at the place of the cleft. Genes or molecules responsible for development of clefts in the lip and palate area can also influence the genes associated with the eruption of the tooth. Similar previous studies demonstrate missing teeth with cleft sides as common. According to this, lateral incisors are key teeth to be missing [11]. The present study also showed the same results with more prevalence of 16 patients who had lateral incisors missing. Other commonly missing teeth in this study included premolars (19 patients) and canines (2 patients). Premolars are directed by another set of genes compared to lateral incisors. The difference in the prevalence of different missing teeth in the two studies indicate that two different set of genes might be altered differently in these two populations. Like other dental deformities mentioned earlier, it is reported that agenesis of teeth is more common near the cleft [21]. In this study there were 38% missing teeth (agenesis), 17% hypodontia (impacted), 8% anodontia (agenesis). Missing teeth either through agenesis or impactions are usually caused by decreased blood supply near the cleft area and also because of deficient mesenchymal mass at the cleft.

Akcam et al. in 2010 showed that 19% of the population with cleft lip and palate had a prevalence of impactions in Turkey [11]. Similarly, this study showed significant p-values for hypodontia associated with CL (p-value: 0.007), CP (0.03), and CLP (0.006). So, this study specifically shows the association of dental anomalies such as hypodontia with different types of orofacial clefts (CL/CP/CLP). Changes in the shape of teeth have been reported in many previous studies. Changes in the shape of teeth may present on cleft as well as non-cleft sides [11]. The present study reports the presence of irregularly shaped teeth in patients with a cleft. The study showed 7% malformed teeth, 8% Hutchinson incisor, 5% microdontia, and 2% macrodontia. These irregular and malformed teeth were not found to be significant and would require a large population-based sample to rule out their normal presence and their association with OFCs.

Following agenesis of teeth, it has been shown in previous studies that supernumerary teeth are also common in patients with clefts [22]. Supernumerary teeth in OFCs are formed due to fragmentation of dental lamina at the site of the cleft. This study also showed 16% of the patients having supernumerary teeth. Previous research showed 1.9% to 10% of supernumerary teeth identified in patients with CP and UCLP. Another study showed a higher incidence of 22.2% supernumerary teeth with UNCLP, CP, and both [23]. Similar to these reports, according to this study, patients with CLP and supernumerary teeth showed highly significant results with a p-value of 0.04. More destruction of soft and hard tissue in patients of CLP could be marked during orofacial development. The abnormal gene functioning such as BMP, FGF signaling may be responsible for increased fragmentation of dental lamina results in the development of supernumerary teeth.

## Conclusions

OFCs in all its three forms are significantly associated with dental irregularities such as missing teeth and supernumerary teeth. During treatment of clefts, the plastic surgeon or maxillofacial surgeon should consider these dental irregularities. The treatment plan should include detailed dental rehabilitation plans. There is a strong need for dentists to be working alongside other doctors to provide better care for patients with an OFC. Potential limitations may include recall bias for the cause of missing teeth. Other limitations may include the presence of such dental anomalies in the normal population.

## References

[1] Kosturos M, Wainwright G, Abumustafa A (2019). IRF6 gene Variants in Etiology of Nonsyndromic Cleft Lip and Palate, and in Syndromes with Orofacial Clefts. https://scholarlycommons.pacific.edu/excellence-day/2019/events/22/.

[2] Mossey P, Little J (2009). Addressing the challenges of cleft lip and palate research in India. Indian J Plast Surg.

[3] Yaqoob M, Mahmood F, Hanif G, Bugvi SM, Sheikh MA (2013). Etiology and genetic factors in clefts of lip and/or palate reported at children's hospital, Lahore, Pakistan. Indian J Hum Genet.

[4] Yazdee AK, Saedi B, Sazegar AA, Mehdipour P (2011). Epidemiological aspects of cleft lip and palate in Iran. Acta Med Iran.

[5] Nanci A (2017). Ten Cate's Oral Histology-e-book: development, structure, and function. https://www.elsevier.com/books/ten-cates-oral-histology/nanci/978-0-323-48524-1.

[6] Lu C, Wang JY, Jia ZL (2019). Environmental factors of non-syndromic cleft lip and palate. Hua Xi Kou Qiang Yi Xue Za Zhi.

[7] Stein RA (2007). Smith's recognizable patterns of human malformation, 6th edition. Arch Dis Child.

[8] Krapels IP, Vermeij-Keers C, Müller M, de Klein A, Steegers-Theunissen RP (2006). Nutrition and genes in the development of orofacial clefting. Nutrition reviews.

[9] Mahamad Irfanulla Khan AN, Prashanth CS, Srinath N (2020). Genetic etiology of cleft lip and cleft palate. AIMS Molecular Science.

[10] Camporesi M, Baccetti T, Marinelli A, Defraia E, Franchi L (2010). Maxillary dental anomalies in children with cleft lip and palate: a controlled study. Int J Paediatr Dent.

[11] Akcam MO, Evirgen S, Uslu O, Memikoğlu UT (2010). Dental anomalies in individuals with cleft lip and/or palate. Eur J Orthod.

[12] Ranta R (1986). A review of tooth formation in children with cleft lip/palate. Am J Orthodont Dentofacial Orthop.

[13] Tortora C, Meazzini MC, Garattini G, Brusati R (2008). Prevalence of abnormalities in dental structure, position, and eruption pattern in a population of unilateral and bilateral cleft lip and palate patients. Cleft Palate Craniofac J.

[14] Dahllöf G, Ussisoo-Joandi R, Ideberg M, Modeer T (1989). Caries, gingivitis, and dental abnormalities in preschool children with cleft lip and/or palate. Cleft Palate J.

[15] Nicot R, Rotten D, Opdenakker Y, Kverneland B, Ferri J, Couly G, Levaillant JM (2019). Fetal dental panorama on three-dimensional ultrasound imaging of cleft lip and palate and other facial anomalies. Clin Oral Investig.

[16] Elahi MM, Jackson IT, Elahi O, Khan AH, Mubarak F, Tariq GB, Mitra A (2004). Epidemiology of cleft lip and cleft palate in Pakistan. Plast Reconstr Surg.

[17] Derijcke A, Eerens A, Carels C (1996). The incidence of oral clefts: a review. Br J Oral Maxillofacial Surg.

[18] Bush JO, Jiang R (2012). Palatogenesis: morphogenetic and molecular mechanisms of secondary palate development. Development.

[19] Demirjian A, Goldstein H, Tanner JM (1973). A new system of dental age assessment. Hum Biol.

[20] Ribeiro LL, das Neves LT, Costa B, Gomide MR (2002). Dental development of permanent lateral incisor in complete unilateral cleft lip and palate. Cleft Palate Craniofac J.

[21] Lourenço Ribeiro L, Teixeira Das Neves L, Costa B, Ribeiro Gomide M (2003). Dental anomalies of the permanent lateral incisors and prevalence of hypodontia outside the cleft area in complete unilateral cleft lip and palate. Cleft Palate Craniofac J.

[22] Al Jamal GA, Hazza'a AM, Rawashdeh MA (2010). Prevalence of dental anomalies in a population of cleft lip and palate patients. Cleft Palate Craniofac J.

[23] Vichi M, Franchi L (1995). Abnormalities of the maxillary incisors in children with cleft lip and palate. ASDC J Dent Child.

